# High-Toughness Poly(lactic Acid)/Starch Blends Prepared through Reactive Blending Plasticization and Compatibilization

**DOI:** 10.3390/molecules25245951

**Published:** 2020-12-16

**Authors:** Huan Hu, Ang Xu, Dianfeng Zhang, Weiyi Zhou, Shaoxian Peng, Xipo Zhao

**Affiliations:** Hubei Provincial Key Laboratory of Green Materials for Light Industry, Collaborative Innovation Center of Green Light-Weight Materials and Processing, Hubei University of Technology, Wuhan 430068, China; huhuan13579@163.com (H.H.); xuang126912@163.com (A.X.); wodiao1066@163.com (D.Z.); zhouweiyi@hbut.edu.cn (W.Z.); psxbb@126.com (S.P.)

**Keywords:** poly(lactic acid), starch, reactive blending, plasticization, compatibilization

## Abstract

In this study, poly(lactic acid) (PLA)/starch blends were prepared through reactive melt blending by using PLA and starch as raw materials and vegetable oil polyols, polyethylene glycol (PEG), and citric acid (CA) as additives. The effects of CA and PEG on the toughness of PLA/starch blends were analyzed using a mechanical performance test, scanning electron microscope analysis, differential scanning calorimetry, Fourier-transform infrared spectroscopy, X-ray diffraction, rheological analysis, and hydrophilicity test. Results showed that the elongation at break and impact strength of the PLA/premixed starch (PSt)/PEG/CA blend were 140.51% and 3.56 kJ·m^−2^, which were 13.4 and 1.8 times higher than those of pure PLA, respectively. The essence of the improvement in the toughness of the PLA/PSt/PEG/CA blend was the esterification reaction among CA, PEG, and starch. During the melt-blending process, the CA with abundant carboxyl groups reacted in the amorphous region of the starch. The shape and crystal form of the starch did not change, but the surface activity of the starch improved and consequently increased the adhesion between starch and PLA. As a plasticizer for PLA and starch, PEG effectively enhanced the mobility of the molecular chains. After PEG was dispersed, it participated in the esterification reaction of CA and starch at the interface and formed a branched/crosslinked copolymer that was embedded in the interface of PLA and starch. This copolymer further improved the compatibility of the PLA/starch blends. PEGs with small molecules and CA were used as compatibilizers to reduce the effect on PLA biodegradability. The esterification reaction on the starch surface improved the compatibilization and toughness of the PLA/starch blend materials and broadens their application prospects in the fields of medicine and high-fill packaging.

## 1. Introduction

With the increasing tension in petroleum resources and environmental issues, several bio-based degradable resources have been drawing wide attention in recent years. Poly(lactic acid) (PLA), which is the most promising bio-based degradable material, can be obtained through the polymerization [1] of lactic acid by fermenting plant starch. PLA has broad application prospects in daily lives, packaging industry, and the biomedical field [2,3,4] due to its good mechanical properties and biocompatibility. However, the high brittleness, low impact strength [5], and high cost of this material limit its development and applications. An effective way to overcome these drawbacks is to add other biodegradable materials to PLA [6]. Blending PLA with starch [7], which is a widely derived biodegradable material, yields high-performance and low-cost PLA/starch blends and expands the application range of renewable resources.

Starch contains numerous hydroxyl groups and has high polarity. However, when starch and PLA are blended, using the former as a filler cannot improve and even reduces the toughness of the PLA/starch blends. The layered microstructure along the flow direction is formed in the PLA/starch blends through the pressure-induced flow (PIF) process proposed by Zhang et al. [8]. During the compression process, PLA is oriented, whereas starch is deformed. This process increases the uniformity of the thickness of the PLA crystal layer. The layered structure allows the PLA/starch blend to absorb high impact energy, thereby improving the toughness of the material. However, the use of PIF to improve the toughness of the material is limited, and high pressure causes material damage. Small molecular plasticizers [9,10], such as polyethylene glycol (PEG) and citrate esters, can improve the ductility of PLA/starch blend materials but cannot change the immiscibility of PLA and starch. Plasticizers migrate with time and do not stabilize in the blends. Starch can be chemically modified through esterification, oxidation, crosslinking, and grafting [11] to promote the compatibility of the PLA/starch blend materials. To weaken the hydrogen bonding force among the starch molecular chains, the starch particle size or the polarity of the starch surface can be reduced to enhance the interaction force between PLA and starch. Wang et al. [12] prepared core–shell starch nanoparticles (CSS NPs). By esterifying the starch and subsequently polymerizing the emulsion, the CSS NPs utilized starch as the core to ensure that the material achieves a strong rigidity and polyethyl acrylate (PEA), which has a good affinity with PLA, as shell. Given the synergistic effect of starch and PEA, the PLA/CSS NP blends exhibited excellent toughness. Thielemans et al. [13] modified nanosized single crystal starch particles by using stearoyl chloride and PEG methyl ether. The crystal structure of the modified starch did not change, but the graft crystallized on the surface of the modified starch did. Appropriate grafts can weaken the hydrogen bonding force among starches and reduce the polarity of the starch surface. These changes help improve the compatibility of PLA/starch blends. In addition to the two-step method to modify PLA/starch composite materials, the chemical modification of starch can also be achieved through one-step reactive blending, and at the same time the compatibility of the PLA/starch composite system can be enhanced. Xiong et al. [14] synthesized a bio-based monofunctional epoxy compound Epicard and used it for the reactive compatibilization of PLA/starch composite materials. The epoxy groups on the Epicard can react with starch and form a hydrophobic layer on the surface of the latter, which will enhance the adhesion of the PLA/starch blends and improve the compatibility between PLA and starch. Jariyasakoolroj et al. [15] used chloropropyl trimethoxysilane (CPMS) modified starch (CP-starch) to melt blend with PLA to prepare PLA/starch composites. During the melt-blending process, CP-starch reacted with PLA to form a block copolymer, which acts as a nucleating agent and can significantly increase the crystallinity of the blend and improve the compatibility between PLA and starch. Modifying starch can effectively improve its surface polarity and increase the interaction force between starch and PLA. Reactive blending, which is a simple and practical method, can directly establish the compatibility of PLA/starch composite materials.

In this study, PLA/premixed starch (PSt)/PEG/citric acid (CA) blends were prepared through reactive blending to examine the effects of CA and PEG on the toughness of PLA/PSt (wt%/wt%, 70/30) blends. CA with high carboxyl underwent an esterification reaction with starch and consequently enhanced the interface adhesion between starch and PLA. As a plasticizer of PLA and starch, PEG effectively dispersed in the matrix and enhanced the mobility of the molecular chains. When the PEG that entered the matrix participated in the esterification reaction between CA and starch, a branched/crosslinked polyester was easily formed at the interface. This polyester further improved the compatibility of the PLA/starch blend materials. This PLA/starch composite material uses biodegradable raw materials, and at the same time, a highly filled PLA/starch composite material with excellent performance is obtained through reactive blending. Therefore, this composite material has broad application prospects in the medical field and packaging field.

## 2. Materials and Methods

### 2.1. Materials

The PLA (2003D; density and melting temperature were 1.24 g/cm^3^ and 160 °C, respectively) was purchased from NatureWorks LLC (Minnetonka, MI, USA). The corn starch (St) was provided by Wuhan Huali Environmental Protection Technology Co., Ltd (Wuhan, Chian). The citric acid (CA) monohydrate and PEG 1000 (analytical purity) were purchased from Sinopharm Chemical Reagent Co., Ltd (Wuhan, Chian). The vegetable oil polyol (VOP) was provided by Dongguan Aoda Environmental New Materials Co., Ltd (Dongguan, Chian).

### 2.2. Preparation of the PLA/PSt/PEG/CA Sample

PLA and starch were placed in a 60 °C vacuum drying oven and dried for 24 h to reduce the moisture content of the latter to approximately 3%. The PSt was prepared by mixing dry starch with VOP for 15 min at a mass ratio of 7:3. The mixtures of PLA, PSt, PEG, and CA were melt-blended at 170 °C with a rotation speed of 60 rpm for 10 min. A blend of different components was obtained, and the corresponding formulations are presented in Table 1. The mixtures were then pressed into plates with thicknesses of 1, 2, and 4 mm by using a plate vulcanizer. These plates were cut and polished into standard splines for testing. The samples were completely dissolved in dichloromethane, centrifuged three times, washed with ethanol to separate the precipitate, and then dried in a vacuum oven at 60 °C for 48 h.

### 2.3. Characterization

#### 2.3.1. Mechanical Property Measurements

The hot pressed 2 mm-thick plate-shaped samples were cut and polished into dumbbell-shaped samples in accordance with the international standard ISO 527-2. The CMT-4204 electric tensile tester (SANS, Shenzhen, Chian) was used to test the samples at a tensile speed of 5 mm/min to obtain the stress–strain curve, tensile strength, and elongation at break. The 4 mm-thick samples were cut into a standard spline in accordance with ISO 179-1, and the impact energy was measured through the Charpy impact test (SJJ-50, Chengde, Chian) with a range of 0.5 J. The impact strength of the sample was obtained using data calculation. The data of the samples in every test were measured on at least five splines.

#### 2.3.2. Rheological Behavior Analysis

A rotating plate rheometer (AR2000EX, TA, Shanghai, Chian) was used to obtain the linear rheological behavior of the mixture with plate–plate geometry. The sample is a 1 mm-thick disc with a diameter of 25 mm. The set temperature was 170 °C, the frequency range was 0.1–100 Hz, and the strain was 0.1%.

#### 2.3.3. Differential Scanning Calorimeter (DSC)

A DSC (Perkin Elmer, DSC 8000, Kunming, Chian) was used for crystallization performance analysis of the PLA/PSt/PEG/CA blend. The sample was heated from 30 to 200 °C at a rate of 10 °C/min and maintained at 200 °C for 3 min to eliminate the previous thermal history and then cooled to 30 °C at a rate of 10 °C/min. The sample was heated again from 30 to 200 °C at the same increase rate. The second heating curve was recorded, and the crystallinity of PLA was calculated as [16]

(1) $$X_{c}=\frac{\Delta H_{m}-\Delta H_{c}}{\Delta H_{f}\omega_{PLA}}\times 100\%$$

where *X_c_* represents the crystallinity of PLA, $\Delta H_{m}$ and $\Delta H_{c}$ are the melting enthalpy and cold crystallization enthalpies of the sample, respectively, and $\Delta H_{f}$ and $\omega_{PLA}$ are the melting enthalpy and PLA mass fraction of 100% crystallized PLA, respectively.

#### 2.3.4. Scanning Electron Microscopy (SEM)

The morphology of the impact fracture surface was examined using a SEM (JSM-6390L V, JEOR, Tokyo, Japan) at an acceleration voltage of 10 kV. The 4 mm-thick sample was broken at room temperature, and the impact fracture surface was washed with ethanol.

#### 2.3.5. X-Ray Diffraction (XRD)

The XRD analyzer from Panalytical B.V. (Empyream, Almelo, NL, USA) was used to test the pretreated powder samples with the target of Cu, test conditions: voltage = 40 kV, current = 30 mA, start angle = 5°, end angle = 40°, and scan step width = 0.02°.

#### 2.3.6. Fourier-Transform Infrared (FTIR) Spectroscopy

The blend was dissolved in dichloromethane (CH_2_Cl_2_), washed with ethanol, and dried to obtain powder samples. The powders were mixed with potassium bromide at a weight ratio of 1:100 and subsequently compressed. Measurements were performed using an FTIR spectrometer (Iso10, Nicolet, Beijing, Chian) with a resolution of 4 cm^−1^ and scan number of 32.

#### 2.3.7. Hydrophilicity Test

The water absorption rate of the test sample was tested in accordance with the plastic water absorption test method (GB1034-98). The blends were cut into small pieces with sizes of 10 × 10 × 2 mm and placed in a vacuum oven at 50 °C for 48 h. The initial weight m_1_ of a piece was measured using an analytical balance. Subsequently, the sample was soaked in deionized water for 24 h, wiped with filter paper, and weighed to determine the final weight m_2_. The water absorption rate of the blend materials was determined as [17].

(2) $$\mathrm{Water}\;\mathrm{absorption}\;=\;\frac{m_{2}-m_{1}}{m_{1}}\times 100\%$$

## 3. Results and Discussions

### 3.1. Mechanical Properties of PLA/PSt/PEG/CA Blends

**Figure 1:**
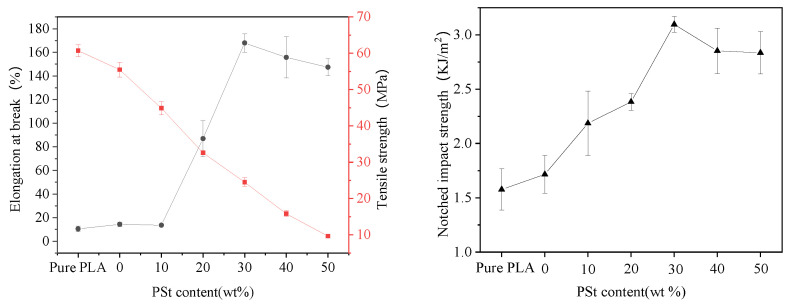
Mechanical properties of pure poly(lactic acid) (PLA) and PLA/premixed starch (PSt)/polyethylene glycol (PEG)/citric acid (CA) blends with different PSt contents.

The mechanical properties of pure PLA and PLA/PSt/PEG/CA blends with different PSt mass ratios are shown in Figure 1. The elongation at break and the notched impact strength of the PLA are 10.45% and 1.58 kJ·m^−2^, respectively, which reflects the brittleness of PLA. Compared with pure PLA, the elongation at break of PLA/PEG/CA blends increased slightly, while the tensile strength decreased, which may be due to the ability of low-molecular-weight PEG to plasticize PLA [18]. There is also an explanation that under the action of high temperature shear, a small amount of water and CA in PEG can promote the depolymerization of PLA [19], and the low-molecular-weight PLA produced by depolymerization can also have a plasticizing effect on PLA [20]. When the PSt content is less than 10wt%, the tensile strength of the blend decreases with the increase in PSt content, which is mainly due to the plasticization of PLA. The change in elongation at break is not obvious, which indicates that a new interaction force is generated between the blended components and a new structure is formed. This structure works together with the plasticized PLA to offset the defects caused by the incompatibility of PLA and starch, and this structure can improve the impact strength of the blend. The elongation at break and the impact strength of the blends show a significant upward trend as the PSt content increases and reaches the maximum at 30 wt%. This phenomenon can be attributed to the increased interaction between the components of the blend. Compared with the plasticizing effect of PEG and CA on PLA, the interaction force between the blending components at this time has a more significant effect on improving the toughness of the PLA/PSt/PEG/CA blend. When the PSt content exceeds 30 wt%, the elongation at break slowly decreases. The excess PSt forms an agglomeration, and concentration of stress occurs during the stretching process of the blend and results in increased defects. Conversely, the tensile strength of the blend monotonically decreases with the increase in the PSt content, which indicates that some components in the blend enhance the mobility of the PLA molecular chain. The results of the impact test are consistent with the trend of the elongation at break. The impact strength of the blend first increased and then decreased with the increase in PSt content, and reached the maximum when the PSt content was 30 wt%, indicating that the adhesion between PLA and starch was the largest at this time. When the PSt content exceeds 30 wt%, the possibility of PSt agglomeration in the blend increases, resulting in a decrease in the mechanical properties of the material. Therefore, a blend with a PSt content of 30 wt% was selected for follow-up research.

To explore the contribution of each component of the PLA/PSt/PEG/CA blend to the mechanical properties of the blend, several blends with different components were fabricated. The test results are summarized in Table 2. Most raw materials blended with PLA alone caused the toughness of the blend to decrease, and only the PLA/PSt/CA blend and PLA/PSt/PEG/CA blend had a sudden change in toughness. This phenomenon indicates that the reason for the improved toughness of PLA/starch composites may be that the PEG and CA in the system improve the interface interaction between PLA and starch. Compared with pure PLA, the tensile strength and elongation at break of the PLA/PSt blends significantly declined. This phenomenon may be due to the weak interaction between the PLA and PSt components, which results in weak tangles among molecular chains and poor compatibility among components. After introducing a small amount of PEG into the PLA/PSt blend, the elongation at break of the new blend slightly increased and the tensile strength decreased. This result can be attributed to the plasticizing effect of PEG. PEG can enhance the mobility of the PLA molecular chain and increase the tensile toughness of the PLA/PSt/PEG blends. However, the PLA/PSt/CA blends showed obvious strain hardening, and their elongation at break reached 54.47%. After introducing PEG, the elongation at break of the PLA/PSt/PEG/CA blend significantly increased to 140.51%. The addition of CA increases the interaction force among the blend components, and at this time the negative effects of CA and water on PLA can be ignored. However, when CA and PEG are present in the blend at the same time, PEG acts more than just a plasticizer in the PLA/PSt/PEG/CA blend. Compared with that of the PLA/PSt/CA blend, the tensile toughness of the PLA/PSt/PEG/CA blend greatly improves. PEG may chemically react with other components during the blending process. This reaction can promote the movement of the PLA molecular chain and improve the compatibility among the blend components.

### 3.2. Rheological Behavior of PLA/PSt/PEG/CA Blends

**Figure 2:**
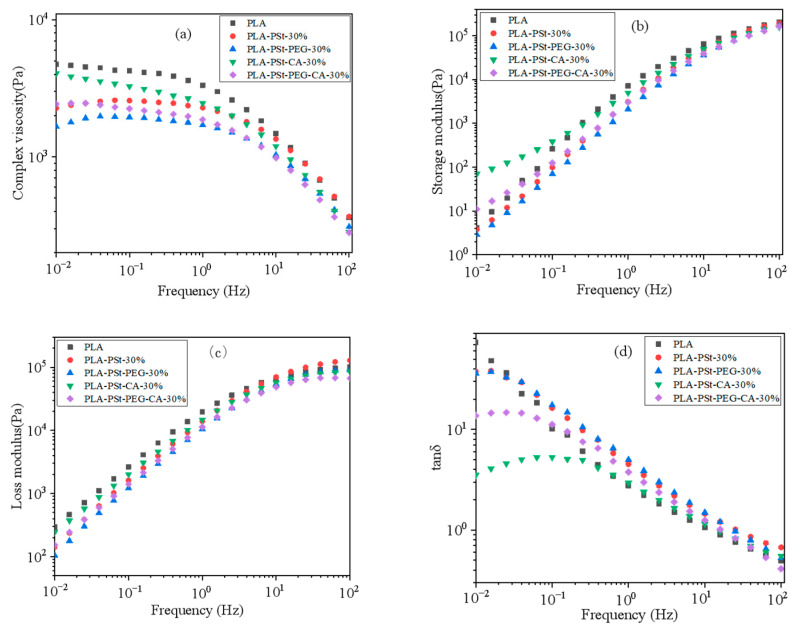
(**a**) Complex viscosity, (**b**) storage modulus, (**c**) loss modulus, and (**d**) loss tangent (tan δ) of PLA and different blends with PSt ratios of 30 wt%.

By testing the rheological properties of the blend, it is helpful to analyze the changes in the compatibility of the blend components. Generally, after PLA is blended with poorly compatible materials such as polyamide11 [21], starch [22], and cellulose [23], the viscosity of the composite material decreases. The introduction of compatibilizers, coupling agents or reactive compatibilizers can increase the interaction force effectively between the interfaces of the components of the composite material, thereby increasing the viscosity of the composite material. The rheological properties of the blends, such as storage modulus, complex viscosity, and loss modulus, were analyzed to explore the effects of PSt, PEG, and CA on the melt strength of the blend. The presence of PSt can change the viscosity and degree of shear-thinning [24] as compared with neat PLA. The dependence of the complex viscosity of different blends on frequency is illustrated in Figure 2a. The figure shows two types of the complex viscosity frequency dependence of the blends. In the low-frequency region, the viscosity of the blends after PSt addition decreases in varying degrees compared with that of pure PLA. Among the blends, the decrease in the viscosity of the PLA/PSt/PEG blends is the most obvious. This result can be attributed to the immiscibility of PLA and starch and the plasticizing effect of PEG on PLA [25]. The poorly compatible blend components are prone to phase separation, forming a defect structure with weak interaction force [26]. When external forces act on the blend, this defect structure cannot absorb energy, resulting in a decrease in the viscosity of the blend. PEG is dispersed among the PLA molecular chains during the blending process, and such dispersion enhances the mobility of the PLA molecular chains and reduces the viscosity [27] of the blend. The viscosity of the blend without CA does not change with the change in the low-frequency region, thereby showing the properties of a Newtonian fluid, as well as an obvious shear thinning in the high-frequency region. The platform of the curve of the viscosity of the CA-containing blend in the low-frequency region disappears, suggesting that CA can increase the shear-thinning strength of the blend. The degree of entanglement among the components of the blend hinders the movement of the PLA molecular chain and therefore results in increased viscosity in the low-frequency region.

The behavior of the storage and loss moduli as a function of frequency is shown in Figure 2b,c, respectively. The storage modulus (G′) of PLA in the low-frequency region is lower than its loss modulus (G”); such a discrepancy implies that PLA shows typical viscous characteristics. This relationship slightly changes after adding PSt and PEG to the blend. When the CA component is added, a significant increase in the storage modulus of the blend is observed in the low-frequency region. This increment indicates that the existence of CA can promote the transformation of PLA from a liquid-like state to a solid-like one [28]. The change in tan δ during frequency scanning is used to estimate the dispersion of the filler or the dispersed phase in the blended system. The tan δ of liquid-like materials linearly decreases with the increase in frequency. The changes in the tan δ of several samples with frequencies at 170 °C are displayed in Figure 2d. The finding shows that PLA has a typical liquid-like characteristic [29] which slightly changes after adding PSt and PEG. After adding CA, the tan δ value of PLA/PSt/CA blend and PLA/PSt/PEG/CA blend in the low-frequency region significantly decreases, which means that a gel-like substance that can help improve the compatibility of PLA and PSt may appear in the system. The frequency dependence of tan δ weakens, indicating that the blend tends to change from a liquid-like state to a solid-like one.

### 3.3. Crystallization Behavior of PLA/PSt/PEG/CA Blends

**Figure 3:**
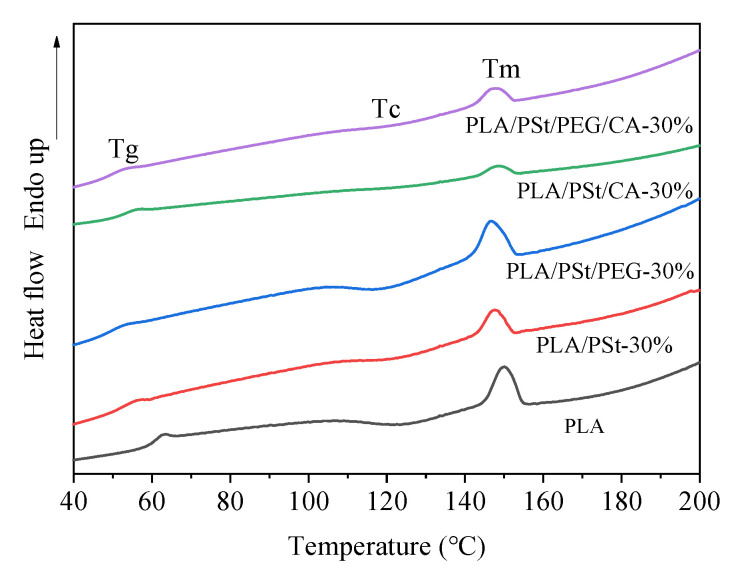
Differential Scanning Calorimeter (DSC) heat flow curves of pure PLA and PLA/PSt, PLA/PSt/PEG, PLA/PSt/CA, and PLA/PSt/PEG/CA blends.

**Figure 4:**
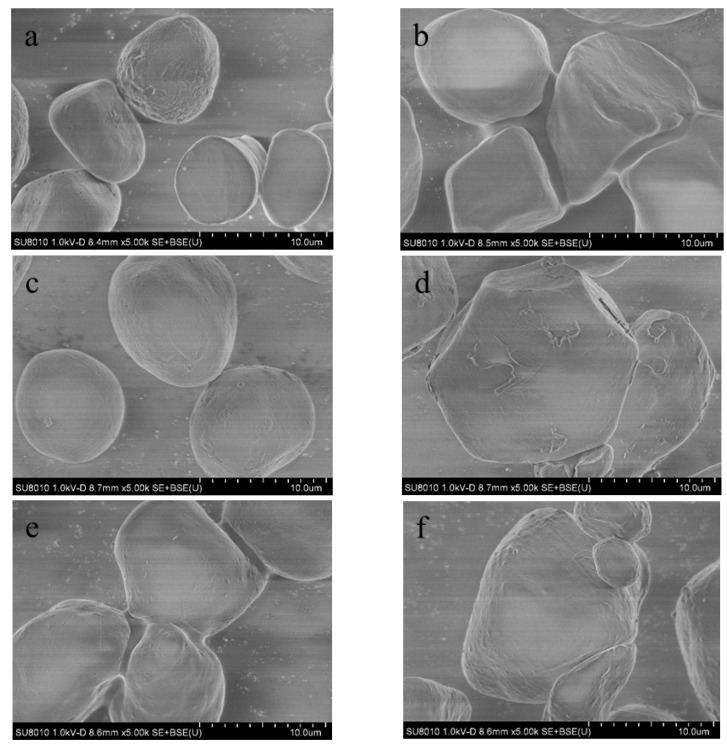
Surface morphology of several pretreated blend powder samples observed using SEM: (**a**) pure starch, (**b**) PSt, (**c**) PLA/PSt, (**d**) PLA/PSt/PEG, (**e**) PLA/PSt/CA, and (**f**) PLA/PSt/PEG/CA.

PLA is a typical semi-crystalline polymer [30] whose mechanical properties are closely related to the thermal properties and crystallinity of a solid. The heat flow curves of several samples during the second temperature rise are shown in Figure 3 and Table 3. Compared with pure PLA, PLA/PSt blends have lower T_g_ and T_c_, which may be due to the VOP entering the PLA matrix during the blending process destroying the crystallization of PLA. Secondly, the VOP attached to the surface of the starch blocks the interface between PLA and starch, inhibiting the heterogeneous nucleation [31] of PLA by starch. The results of analysis of mechanical properties and rheological behavior have explained the immiscibility of PLA and PSt. The crystallinity of PLA in the PLA/PSt blend calculated by formula (1) is reduced, which also confirms the reliability of the analysis. The T_g_, T_c_, and T_m_ of PLA/PSt/PEG blends further decreased, which was mainly due to the plasticization of PLA by PEG. PEG can enter between PLA macromolecules during melt mixing and establish physical interactions such as hydrogen bonding or dipole-dipole interactions in between atoms. As a result, some of the rigid homogeneous PLA-PLA interactions were replaced by heterogeneous PLA-PEG interactions. This phenomenon enhanced the mobility of PLA and decreased the energy consumption during glass transition (decreased T_g_) [32]. Compared with the PLA/PSt blend, the T_c_ and T_m_ of the PLA/PSt/CA blend increased. This may be because the surface polarity of the starch changed after CA was introduced into the blend. The reason for the change may be that CA participated in the esterification reaction and formed a branched/crosslinked copolymer on the surface of the starch, which enhanced the interaction between the components of the blend [33]. Usually, T_g_ will increase as the interaction force between the components of the blend increases. However, the T_g_ of PLA/PSt/CA blends only increased slightly, which may be because CA tends to change the surface polarity of starch rather than acting on PLA. Moreover, the depolymerization of PLA by CA will destroy the crystallization of PLA and reduce X_c_. Comparing PLA/PSt blends with PLA/PSt/CA blends, the result is that CA can slightly increase the T_g_ of the blends. However, when the PLA/PSt/PEG blend is compared with the PLA/PSt/PEG/CA blend, the result is that CA slightly reduces the T_g_ of the blend. This shows that when CA and PEG are contained in the blend, PEG may also participate in the esterification reaction in the melt-blending process. Some interesting facts have also been discovered from the perspective of crystallinity changes in Figure 4. After introducing PEG into the PLA/PSt blend, its crystallinity value increased by 4.32%; however, after introducing PEG into PLA/PSt/CA blend, its crystallinity value only increased by 2.05%. This may be because when the CA component is present in the system, CA restricts the dispersion of PEG in the PLA matrix, thereby attracting PEG to the CA side. Since PEG can plasticize PLA, the branched/crosslinked polymer containing PEG formed after the esterification reaction can more easily penetrate into the PLA matrix and form an amphiphilic bridging structure [34]. This structure is considered to be the “bridge” between PLA and starch, which can enhance the compatibility between PLA and starch. However, a part of the free branched/crosslinked polyester participates in the process of plasticizing [35] PLA and causes the T_g_ of the blend to decrease. The change of the interface structure between PLA and starch indicates that the compatibility of the two is increased. At this time, PSt can be regarded as a heterogeneous nucleating agent of PLA and promotes the crystallization of the PLA matrix. The increase in effective nucleation sites in the blend enhances the interfacial adhesion between PLA and starch and allows the blend exhibit excellent toughness in the macroscopic view, and this has been confirmed from the mechanical performance test results.

### 3.4. Morphology of the Dispersed Phase

On the basis of the above analysis, CA is an important factor in toughening PLA/PSt/PEG/CA blends. To observe the effect of CA and PEG on the blend, a separate dispersed phase of PLA/PSt/PEG/CA blends was obtained by dissolving and washing. The surface morphology of the dried dispersed phase was observed using SEM. The morphologies of several samples examined under 5k× magnification are shown in Figure 4. Pure starch (Figure 4a) appears as an irregular sphere with a diameter of approximately 10 μm and has many small pits on the surface. As shown in Figure 4b, the surface of PSt is smooth. The starch in the pretreated PLA/PSt blends (Figure 4c) blend has similar size and surface morphology to pure starch. This similarity indicates that the compatibility between PLA and starch is still poor. Figure 4d shows that the size of starch particles in the PLA/PSt/PEG blend after the pretreatment increases to some extent, which may be due to the plasticizing effect of PEG on starch. The presence of numerous fine pits on the surface of starch (Figure 4d) indicates that PLA and starch are not effectively entangled, and starch only acts as a large particle filler in the PLA/PSt and PLA/PSt/PEG blends to reduce their macroscopic mechanical properties. However, although the particle size of starch in the PLA/PSt/CA blend does not significantly change after the addition of CA, the corresponding surface morphology greatly varies and several tiny protrusions form on the surface of the starch granules (Figure 4e). This phenomenon causes the agglomeration of starch, which is also evident in Figure 4f. In addition, the starch particles in Figure 4f seem to be affected by the plasticization of PEG, and the size of the starch particles increases to some extent. The addition of PEG promotes the plasticization of the PLA and starch phases, whereas the addition of CA enhances the compatibility among the components of the blend. The change of the starch surface structure improves the interface compatibility between PLA and starch, thereby increasing the effective nucleation sites in the blend. At this time, PSt can be regarded as a heterogeneous nucleating agent of PLA, which enhances the interfacial adhesion between PLA and starch. The blends show excellent toughness in the macroscopic view, which has been confirmed from the mechanical performance test results.

### 3.5. Crystallization of the Dispersed Phase

**Figure 5:**
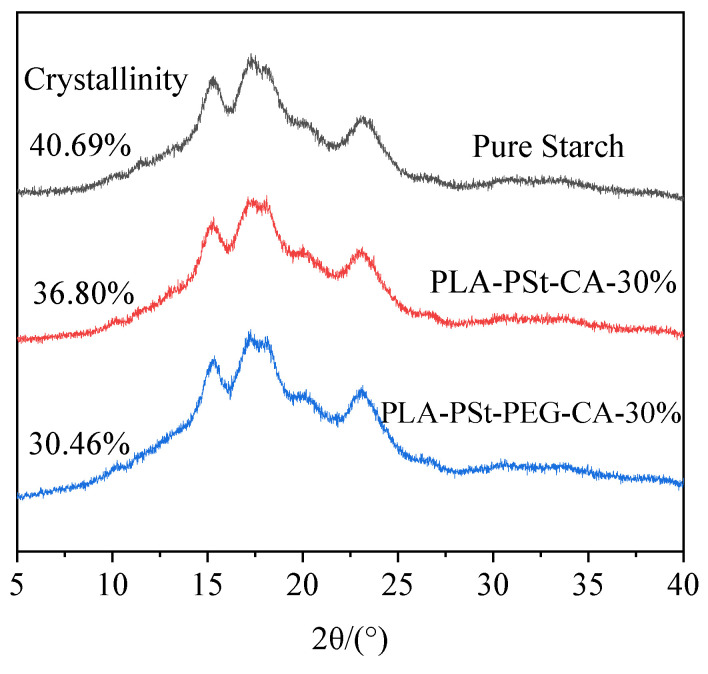
XRD patterns of pure starch and pretreated PLA/PSt/CA and PLA/PSt/PEG/CA powder samples.

The XRD patterns of pure starch and pretreated PLA/PSt/CA and PLA/PSt/PEG/CA powder samples are shown in Figure 5. The shape of the XRD diffraction peaks of the three samples does not significantly change, which means that the crystalline type [36] of starch in the samples does not vary. The crystallinity of the three samples was obtained by fitting the XRD data. The crystallinity of the samples containing CA decreases compared with that of pure starch because the former destroys the amorphous area of starch to a certain extent, thereby enhancing the connection between PLA and the starch molecular chains and the compatibility between PLA and starch. In addition, the crystallinity of the blend further decreases after adding PEG, which suggests that PEG not only acts on PLA, but also plasticizes starch [37] to a certain extent. During the blending process, the crystalline area of starch is partly destroyed by the PEG and CA. This phenomenon facilitates the plasticization of starch and enhances starch dispersibility in PLA, and thus improves the compatibility between PLA and starch.

### 3.6. FTIR Analysis of the Dispersed Phase

**Figure 6:**
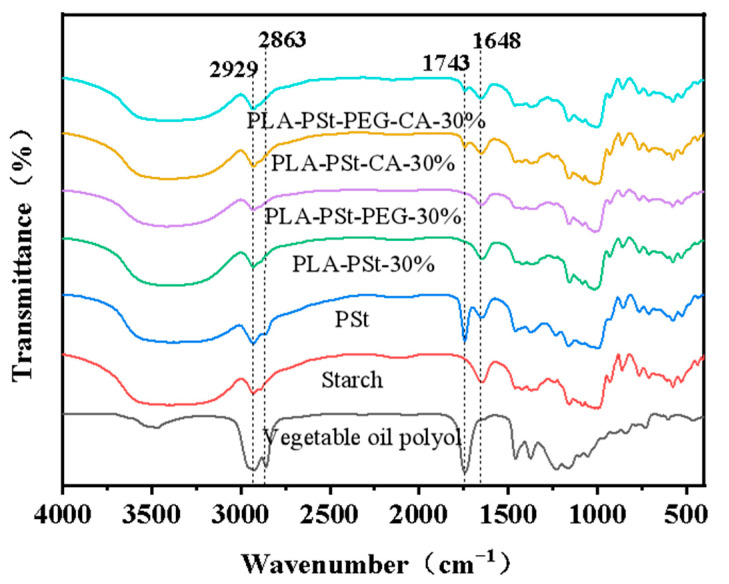
FTIR spectra of vegetable oil polyols, starch, PSt, and pretreated PLA/PSt, PLA/PSt/PEG, PLA/PSt/CA, and PLA/PSt/PEG/CA blend powder samples.

Chemical reactions may affect the surface morphology of starch, and this effect will influence the compatibility of the components of the blend. The dispersed phase was extracted from several blends, and FTIR spectroscopy was performed to analyze the changes in the compatibility among the components. The FTIR test results of several samples are presented in Figure 6. The PLA/PSt, PLA/PSt/PEG, PLA/PSt/CA, and PLA/PSt/PEG/CA blends are powder samples obtained through pretreatment. As shown in the figure, the C=O absorption peak near 1743 cm^−1^ can only be detected in VOP and PSt. No C=O absorption peak is observed in the FTIR spectra of the PLA/PSt and PLA/PSt/PEG powder samples after pretreatment. This result indicates that the PLA and VOP in the blend are completely removed, and PEG may display a plasticizing effect. However, C=O absorption peaks are detected in the PLA/PSt/CA and PLA/PSt/PEG/CA powder samples after pretreatment, which means that ester groups are still present in the powder samples at this time. CA participates in the esterification reaction during the blending process, and a branched/crosslinked copolymer forms. This branched/crosslinked copolymer is coated on the surface of the starch to prevent the removal of the ester group during the blending process. Another obvious change in peak position occurs near 2863 cm^−1^, which can be attributed to the C–H symmetric stretching vibration absorption peak in VOP and PSt. The respective absorption peaks of pretreated PLA/PSt and PLA/PSt/PEG powder samples at 1743 and 2863 cm^−1^ disappear, implying that no chemical changes occur during the blending process. Starch only acts as a large-sized filler, and PEG acts as a plasticizer. The immiscibility between PLA and starch decreases the macroscopic tensile toughness of the blend. The esterification reaction might occur in the PLA/PSt/CA and PLA/PSt/PEG/CA powder samples after pretreatment, which might improve the surface activity of starch and increase the compatibility between PLA and starch. Furthermore, the blend shows a significant increase in tensile and impact toughness.

### 3.7. Hydrophilicity Test of PLA/PSt/PEG/CA Blends

**Figure 7:**
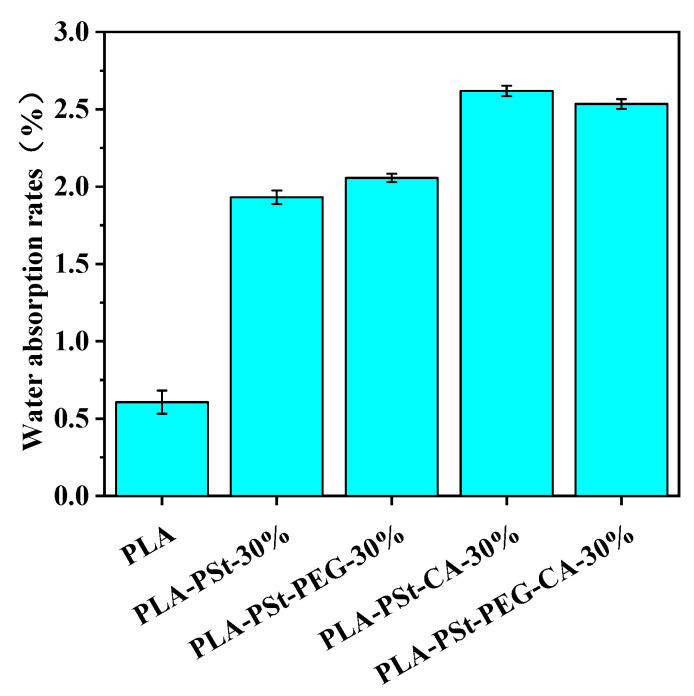
Water absorption rates of pure PLA and PLA/PSt, PLA/PSt/PEG, PLA/PSt/CA, and PLA/PSt/PEG/CA blends.

The water absorption rates of several blends are shown in Figure 7. The addition of hydrophilic starch to PLA improves the water absorption rate of the blend materials. On this basis, this rate will be further improved by adding PEG or CA. These materials contain a large amount of hydrophilic -OH, which helps the blend to combine with H_2_O molecules to form hydrogen bonds. From the results shown in Figure 7, PEG in the blend system seems to have other functions besides acting as a plasticizer. The water absorption rates of the PLA/PSt/CA and PLA/PSt/PEG/CA blends suggest that the hydrophilicity of the blend material decreases after the addition of hydrophilic PEG. This finding reveals that PEG may also participate in the esterification reaction along with CA during the reactive blending process. The polarity of PEG decreases and the content of hydrophilic groups decreases after the esterification reaction after the esterification reaction, resulting in a certain decrease in the water absorption rate of the blend. Not only that, under the combined action of PEG and CA, the interface between PLA and starch becomes more stable, and the interfacial gap becomes smaller, which results in starch not being able to be swollen fully. Secondly, while the crystalline and amorphous regions of starch are destroyed, PEG and CA will be dispersed near the active hydroxyl groups [38] of the starch, rendering these locations unable to accommodate more water molecules and reducing the water absorption of the blend.

**Figure 8:**
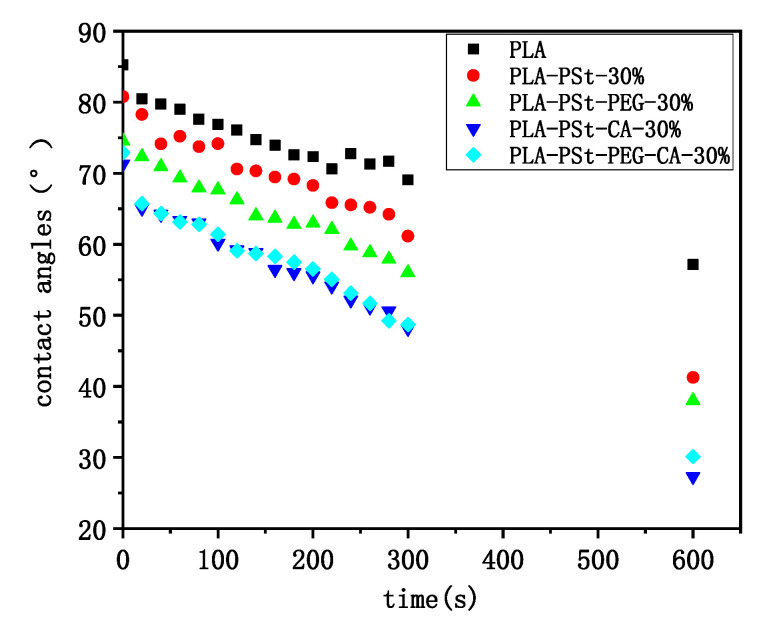
Dynamic contact angles of water in the first 10 min on the surface of pure PLA and PLA/PSt, PLA/PSt/PEG, PLA/PSt/CA, and PLA/PSt/PEG/CA blends.

In view of the change of the hydrophilicity of the blends, the dynamic contact angles of several blends were analyzed. As shown in Figure 8, the contact angle of pure PLA is the largest in the initial state, indicating that PLA is relatively hydrophobic, which is determined by the structural properties of the macromolecular PLA itself. After introducing PSt, PEG and CA into PLA, the contact angles of the blends all decreased, which indicates that the blends are more hydrophilic. However, the contact angle of the PLA/PSt/PEG/CA blend increased compared with the PLA/PSt/CA blend, which is consistent with the conclusion of water absorption. This change further indicates that PEG may participate in the esterification reaction in the PLA/PSt/PEG/CA blend system.

In addition, low-molecular-weight PEG acts as a plasticizer to PLA and exerts a certain plasticizing effect on starch. This feature of PEG allows the branched/crosslinked copolymer with PEG chains that formed after the esterification reaction to penetrate the interface between PLA and starch and act as a bridge between the two to increase their interaction force. Meanwhile, the branched/crosslinked polyester forms a highly stable coating on the starch surface, which facilitates the compatibilization between PLA and starch.

## 4. Conclusions

In this study, the compatibilization between PLA and starch was improved through reactive blending, and the toughness of the PLA/PSt/PEG/CA blend was improved to a certain extent. The elongation at break of the blend with a PSt content of 30 wt% reached a maximum value of 140.51%, and the impact strength increased to 3.56 kJ/m^2^. The addition of a small amount of CA did not change the crystal form of starch, but CA participated in the esterification reaction during the blending process. The generated crosslinked/branched polyester adhered to the surface of the starch granules and improved the surface activity of starch and the compatibility between starch and PLA. The particle size and shape of the starch in the blend did not significantly change, which indicated that the esterification reaction only occurred in the amorphous region of starch. The decrease in the hydrophilicity and the increase in the tensile toughness of the PLA/PSt/PEG/CA blend compared with that of PLA/PSt/CA suggested that PEG may also participate in the esterification reaction during melt blending.

The findings suggest that PEG, which acts as a plasticizer for PLA and starch, can lubricate the system. Moreover, when PEG is dispersed in the PLA and starch, the branched/crosslinked polyester containing PEG chains, which is formed by the esterification reaction, is easily embedded in the interface between PLA and starch. This polyester enhances the interaction between PLA and starch. These results indicate that PEG can form a highly stable crosslinked/branched polyester on the starch surface after participating in the esterification reaction to further improve the compatibility between PLA and starch. Furthermore, the decrease in the water absorption can help enhance the stability of the blend material in a natural environment.

## Figures and Tables

**Table 1:** Material formulations.

	Material	PLA (wt%)	PSt (wt%)	PEG (wt%)	CA (wt%)	St (wt%)	VOP (wt%)
Sample							
1		100	-	-	-	-	-
2		100	-	1.19	0.17	-	1.19
3		90	10	1.19	0.17	10	1.19
4		80	20	1.19	0.17	20	1.19
5		70	30	1.19	0.17	30	1.19
6		60	40	1.19	0.17	40	1.19
7		50	50	1.19	0.17	50	1.19
8		70	30	-	-	30	-
9		70	30	1.19	-	30	1.19
10		70	30	-	0.17	-	-
11		100	-	1.19	-	-	-
12		100	-	-	0.17	-	-
13		70	-	-	-	30	-
14		100	-	-	-	-	5
15		70	-	1.19	-	30	-
16		70	-	-	0.17	30	-
17		100	-	-	0.17	-	5
18		70	-	1.19	0.17	30	-

**Table 2:** Mechanical parameters of different blends.

Materials	Massratio	Tensile Strength/MPa	Elongation at Break/%	Notched Impact Strength/kJ·m^−2^
Pure PLA	100	70.64 ± 2.5	10.45 ± 2.44	2.02 ± 0.15
PLA/PSt	70/30	45.65 ± 3.27	5.04 ± 0.12	2.08 ± 0.12
PLA/PSt/PEG	70/30/1.19	43.5 ± 1.22	7.15 ± 1.28	2.20 ± 0.05
PLA/PSt/CA	70/30/0.17	28.86 ± 2	54.47 ± 9.87	3.18 ± 0.01
PLA/PSt/PEG/CA	70/30/1.19/0.17	28.58 ± 1.38	140.51 ± 10.41	3.56 ± 0.06
PLA/PEG	100/1.19	65.6 ± 3.7	7.6 ± 0.67	-
PLA/CA	100/0.17	65.41 ± 2.76	8.88 ± 0.76	-
PLA/St	70/30	55.08 ± 4.9	5.55 ± 0.17	-
PLA/VOP	100/5	66.52 ± 2.39	10.41 ± 0.95	-
PLA/St/PEG	70/30/1.19	49.84 ± 2.33	4.41 ± 0.31	-
PLA/St/CA	70/30/0.17	47.66 ± 1.69	4.20 ± 0.19	-
PLA/CA/VOP	100/0.17/5	66.78 ± 0.64	7.5 ± 0.45	-
PLA/St/PEG/CA	70/30/1.19/0.17	33.48 ± 5.14	3.43 ± 0.67	-

**Table 3:** Thermal properties of the materials.

Sample Code	T_g_	T_c_	ΔH_c_	T_m_	ΔH_m_	X_c_
	(°C)	(°C)	(J/g)	(°C)	(J/g)	(%)
PLA	62.68	123.63	−9.07	150.05	13.09	6.14
PLA-PSt-30%	55.92	121.95	−5.69	147.66	7.85	3.30
PLA-PSt-PEG-30%	52.97	119.05	−9.94	146.54	15.04	7.78
PLA-PSt-CA-30%	55.98	126.14	−2.11	148.23	3.14	1.57
PLA-PSt-PEG-CA-30%	52.80	123.24	−3.64	147.01	6.01	3.62
